# Isolation of the *phe*-operon from *G. stearothermophilus *comprising the phenol degradative *meta*-pathway genes and a novel transcriptional regulator

**DOI:** 10.1186/1471-2180-8-197

**Published:** 2008-11-13

**Authors:** Bastian Omokoko, Uwe K Jäntges, Martin Zimmermann, Monika Reiss, Winfried Hartmeier

**Affiliations:** 1 Department of Biotechnology, RWTH Aachen University, Worringer Weg 1, 52074 Aachen, Germany; 2 Department of Biology IV (Microbiology & Genetics), RWTH Aachen University, Worringer Weg 1, 52074 Aachen, Germany

## Abstract

**Background:**

*Geobacillus stearothermophilus *is able to utilize phenol as a sole carbon source. A DNA fragment encoding a phenol hydroxylase catalyzing the first step in the *meta*-pathway has been isolated previously. Based on these findings a PCR-based DNA walk was performed initially to isolate a catechol 2,3-dioxygenase for biosensoric applications but was continued to elucidate the organisation of the genes encoding the proteins for the metabolization of phenol.

**Results:**

A 20.2 kb DNA fragment was isolated as a result of the DNA walk. Fifteen open reading frames residing on a low-copy megaplasmid were identified. Eleven genes are co-transcribed in one polycistronic mRNA as shown by reverse transcription-PCR. Ten genes encode proteins, that are directly linked with the *meta*-cleavage pathway. The deduced amino acid sequences display similarities to a two-component phenol hydroxylase, a catechol 2,3-dioxygenase, a 4-oxalocrotonate tautomerase, a 2-oxopent-4-dienoate hydratase, a 4-oxalocrotonate decarboxylase, a 4-hydroxy-2-oxovalerate aldolase, an acetaldehyde dehydrogenase, a plant-type ferredoxin involved in the reactivation of extradiol dioxygenases and a novel regulatory protein. The only enzymes missing for the complete mineralization of phenol are a 2-hydroxymuconic acid-6-semialdehyde hydrolase and/or 2-hydroxymuconic acid-6-semialdehyde dehydrogenase.

**Conclusion:**

Research on the bacterial degradation of aromatic compounds on a sub-cellular level has been more intensively studied in gram-negative organisms than in gram-positive bacteria. Especially regulatory mechanisms in gram-positive (thermophilic) prokaryotes remain mostly unknown. We isolated the first complete sequence of an operon from a thermophilic bacterium encoding the *meta*-pathway genes and analyzed the genetic organization. Moreover, the first transcriptional regulator of the phenol metabolism in gram-positive bacteria was identified. This is a first step to elucidate regulatory mechanisms that are likely to be distinct from modes described for gram-negative bacteria.

## Background

*Geobacillus stearothermophilus*, formerly *Bacillus stearothermophilus*, is a thermophilic, gram-positive, spore-forming bacterium. The rod-shaped organism has an optimal growth temperature of 50–60°C and is widely distributed in soil and sediments. The first strain able to metabolize various phenols was isolated by Buswell and Twomey [1]. Other phenol-degrading bacteria of the same genus isolated since include *G. thermoleovorans *[2], *G. thermoglucosidasius *[3], *G. subterraneus *and *G. uzenensis *[4]. The ability to metabolize phenols is relatively wide spread among the bacterial kingdom. It is thought that this is due to the ubiquitously present plant polyphenols. In particular pseudomonads have been intensively studied on a cellular and sub-cellular level. Findings on the genetic organization of the TOL plasmid encoded *xyl*-genes of *P. putida *mt-2 and the *dmp*-operon of *P. putida *sp. strain CF600 contributed significantly to our current understanding of the aerobic bacterial breakdown of aromatic compounds [5,6]. Less effort has been directed towards gram-positive and thermophilic bacteria. Very little is known concerning the transcriptional regulation.

**Figure 1:**
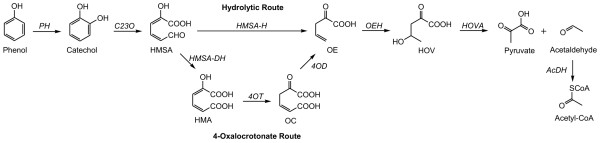
**Phenol degradative *meta*-pathway**. Depending on the substitution, the phenolic compounds are degraded either via the hydrolytic or the 4-oxalocrotonate route. 4OD, OC decarboxylase; 4OT, OC tautomerase; AcDH, acetaldehyde dehydrogenase; C23O, catechol 2,3-dioxygenase; HMA, 2-hydroxymuconic acid; HMSA, 2-hydroxymuconic acid-6-semialdehyde; HMSA-DH, HMSA dehydrogenase; HMSA-H, HMSA hydrolase; HOV, 4-hydroxy-2-oxovalerate; HOVA, HOV aldolase; OC, 4-oxalocrotonate; OE, 2-oxopent-4-dienoate; OEH, OE hydratase; PH, phenol hydroxylase.

The aerobic degradation of aromatic compounds in general can be divided into three basic steps: (i) activation of the aromatic ring, (ii) ring cleavage, (iii) breakdown of the cleavage products to Krebs cycle intermediates. Phenol is converted to catechol in the first step by phenol hydroxylase (PH) which is then subject to either intradiol or extradiol fission of the aromatic ring to yield muconic acid or 2-hydroxymuconic acid-6-semialdehyde (HMSA), respectively. The extradiol cleavage catalyzed by catechol 2,3-dioxygenase (C23O) and the subsequent breakdown to pyruvate and acetyl-CoA is referred to as the *meta*-pathway (Figure 1). HMSA is converted to 2-oxopent-4-dienoate (OE) in three steps via the 4-oxalocrotonate route catalyzed by HMSA dehydrogenase (HMSA-DH), 4-oxalocrotonate tautomerase (4OT) and 4-oxalocrotonate decarboxylase (4OD), respectively, or in one step via the hydrolytic route catalyzed by HMSA hydrolase (HMSA-H). OE is then hydroxylated by OE hydratase (OEH) to yield 4-hydroxy-2-oxovalerate (HOV), which in turn is converted by HOV aldolase (HOVA) to pyruvate and acetaldehyde. Pyruvate can enter the Krebs cycle without any further modifications, acetaldehyde after conversion to acetyl-CoA by acetaldehyde dehydrogenase (AcDH).

As pointed out, phenol can be converted to OE via both the hydrolytic and the 4-oxalocrotonate branch in principle. However, different routes are preferred by different phenolic compounds: phenol and 4-methylphenol enter the oxalocrotonate route, 2- and 3-methylphenol follow the hydrolytic route [5]. Moreover, the cleavage product of 2- and 3-methylphenol (after conversion to 3-methylcatechol) is a ketone that cannot be further oxidized and therefore has to be mineralized via the hydrolytic branch. To our knowledge, the alternative *ortho*-pathway has not been described in thermophilic bacteria yet. No sequences of thermophilic catechol 1,2-dioxygenases triggering the *ortho*-pathway are deposited in public protein databases. However, intradiol cleavage was suggested for the degradation of naphthalene by certain geobacilli [7,8].

*G. stearothermophilus *DSM6285 was isolated as strain BR219 from river sediment [9]. It can grow on phenol at concentrations up to 15 mM. Optimal growth was observed with 10 mM. Other phenolic substrates were not tested. The degradative genes are proposed to reside on the low-copy megaplasmid pGGO1 though these findings are based on phenotypic observations after curing the plasmid from the strain [10]. The same authors suggested the occurrence of a chromosomally encoded *ortho*-pathway, too. In further investigations the cloning of a 2.1 kb DNA fragment and the identification of *pheA *encoding a PH, the partial sequence of *pheB *encoding a C23O and *orfR *encoding a protein of unknown function has been reported [11]. An amperometric biosensor was designed based on the native PH [12]. We conducted a DNA walk in 5' and 3' direction in order to identify all genes of the *meta*-pathway in *G. stearothermophilus*, particularly the C23O-encoding *pheB *which is of primary interest for biosensoric applications. Fifteen open reading frames (ORFs) were identified on a 20.2 kb DNA fragment, ten are predicted to be directly associated with the phenol metabolism. The extrachromosomal localization as well as the operon structure was verified.

## Results

### Metabolism of phenolic substrates

**Figure 2:**
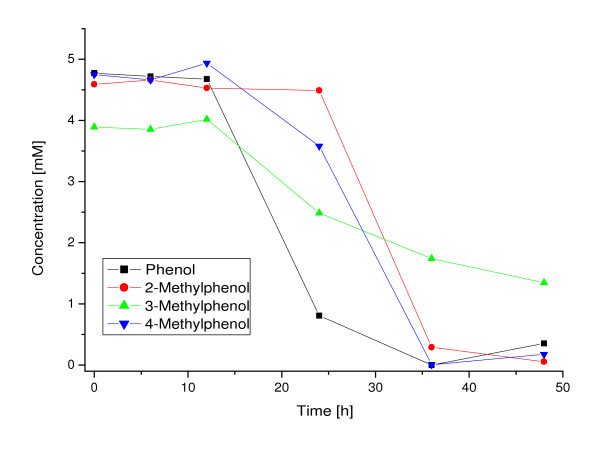
**Growth of *G. stearothermophilus *with different phenolic carbon sources**. Residual concentrations were determined in the medium over a period of 48 h.

*G. stearothermophilus *was grown on minimal media with different phenolic substrates to assess the metabolic versatility. The residual concentration of the aromatic compounds was determined over a period of 48 h by HPLC. As shown in Figure 2, phenol as well as 2-, 3- and 4-methylphenol were metabolized. Cell growth was verified by photometric and microscopic analyses. The findings indicate, that both branches of the *meta*-pathway (2- and 3-methylphenol via hydrolytic route; phenol and 4-methylphenol via 4-oxalocrotonate route) are present in *G. stearothermophilus*.

### PCR-based DNA walk and sequence analysis

**Figure 3:**
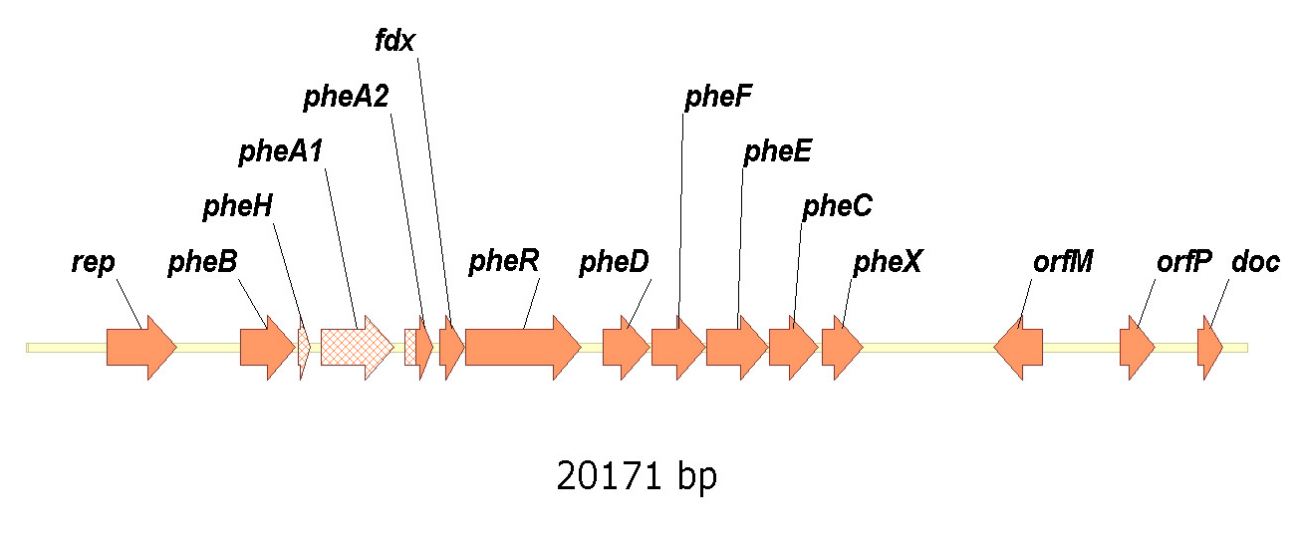
**Schematic representation of the 20.2 kb DNA fragment**. The DNA walk was started from a previously described 2.1 kb fragment (shaded). Fifteen ORFs were identified. The *phe*-genes including *fdx *are associated with the phenol degradative *meta*-pathway.

In order to resolve the genetic organization of the *meta*-pathway genes of *G. stearothermophilus *we performed a PCR-based DNA walk starting out from a previously described 2.1 kb fragment [11]. As a result a DNA fragment with a total length of 20,171 bp was identified. The sequence is available under [GenBank: DQ146476]. Further analysis revealed 15 ORFs (Figure 3). Functions based on similarity could be attributed to 13 ORFs. These are referred to as genes in the following. The function of two ORFs remains unclear. A summary including the length, theoretical molecular mass and coordinates are presented in Table 1. The first base of the start codon of *phe*B was defined as position +1.

**Table 1:** ORFs identified on a 20.2 kb fragment with the positions given relative to *pheB*, the predicted functions based on a similarity search and the molecular mass calculated from the deduced amino acid sequences

ORF	Position [bp]	Size [AS]	Mass [kDa]	Predicted Function
*Rep*	-2211 – (-1053)	386	45.7	Plasmid replication initiator
*pheB*	1 – 912	304	34.7	Catechol 2,3-dioxygenase
*pheH*	951 – 1154	68	7.4	4-Oxalocrotonate tautomerase
*pheA1*	1340 – 2539	400	44.2	Phenol hydroxylase; Monooxygenase subunit
*pheA2*	2730 – 3184	152	16.9	Phenol hydroxylase; Flavin reductase subunit
*fdx*	3306 – 3707	134	14.9	Ferredoxin
*pheR*	3722 – 5647	642	74.3	Regulator
*pheD*	6011 – 6778	256	34.8	2-Oxopent-4-dienoate hydratase
*pheF*	6820 – 7701	294	36.7	Acetaldehyde dehydrogenase
*pheE*	7720 – 8745	342	36.7	4-Hydroxy-2-oxovalerate aldolase
*pheC*	8759 – 9559	267	28.9	4-Oxalocrotonate decarboxylase
*pheX*	9626 – 10303	226	25.8	Carboxylesterase/phospholipase
*orfM*	13269 – 12463	269	31.3	Unknown
*orfP*	14569 – 15129	187	21.5	Unknown
*doc*	15837 – 16256	140	16.5	Death-on-Curing; genetic addiction

### The *phe*-genes encode proteins of the *meta*-pathway

The *phe*-genes including *fdx *are organized in an operon (see below) and except for *pheX *are directly associated with the phenol degradative *meta*-pathway. *PheH*, *pheA1 *and the 5'-end of *pheA2 *were previously identified by Kim and Oriel [11] as *orfR, pheA *and *pheB*. They were thought to encode a protein of unknown function, a PH and a C23O, respectively. Our results indicate that *pheH *encodes a 4OT. *PheA1 *and *pheA2 *encode the monooxygenase and flavin reductase subunits of a two-component PH, similar to a PH from *G. thermoglucosidasius *A7 [2]. A C23O (PheB) was identified immediately upstream of *pheH*. The enzyme belongs to the class of extradiol dioxygenases that are inactivated upon oxidation of their Fe^2+^-cofactor. Cofactor regeneration is mediated *in vivo *by plant-type ferredoxins [13]. Our findings suggest that *fdx *encodes a protein with high similarities to bacterial ferredoxins involved in the reductive reactivation. The deduced amino acid sequence exhibits several features exclusively found in this class of ferredoxins, for example the predomoinance of basic over acidic amino acids resulting in a net positive charge at pH 7 [14]. Four other genes (*pheD, pheF*, *pheE, pheC*) encode enzymes (OEH, AcDH, HOVA, 4OD) that play a direct catalytic role in the *meta*-pathway. The proteins show sequence identities ranging from 59.9% up to 80% to homologous mainly from geobacilli and bacilli.

*PheR *is the largest ORF with 1926 bp. The deduced amino acid sequence displays similarities to transcriptional regulators mostly associated with phenol-metabolism. The protein is larger in comparison to homologoues from gram-negative bacteria and represents the first transcriptional regulator of the phenol metabolism identified in gram-positive bacteria. The architecture of the sub-domains shows significant differences to DmpR and XylR from *P. putida *[15]. Overall, PheR displays the highest similarities (19.6% sequence identity/29.4% sequence similarity) to a σ^54^-specific transcriptional regulator from *Geobacter lovleyi *SZ (GenBank ZP_01594255).

The last gene of the *phe*-operon is *pheX*, which encodes a protein that displays similarities to carboxylesterases and phospholipases. The lipase active site consensus pentapeptide [GXSXG] spans amino acids 110 to 114 of the translated protein sequence [16]. A direct role of an esterase in the phenol breakdown is not apparent. However, the active site consensus sequence can also be found in several HMSA-Hs including XylF from *P. putida *and PheC from *G. thermoglucosidasius*. Whether PheX represents a novel HMSA-H remains subject to further investigations but is doubtful. The enzyme displays only low sequence homologies (<15%/<25%) to both XylF and PheC, which in turn share 28.5% sequence identity and 40.6% sequence similarity. Nevertheless, *pheX *is co-transcribed with the other *phe*-genes (see below).

### Identification of a truncated HMSA-H

**Figure 4:**
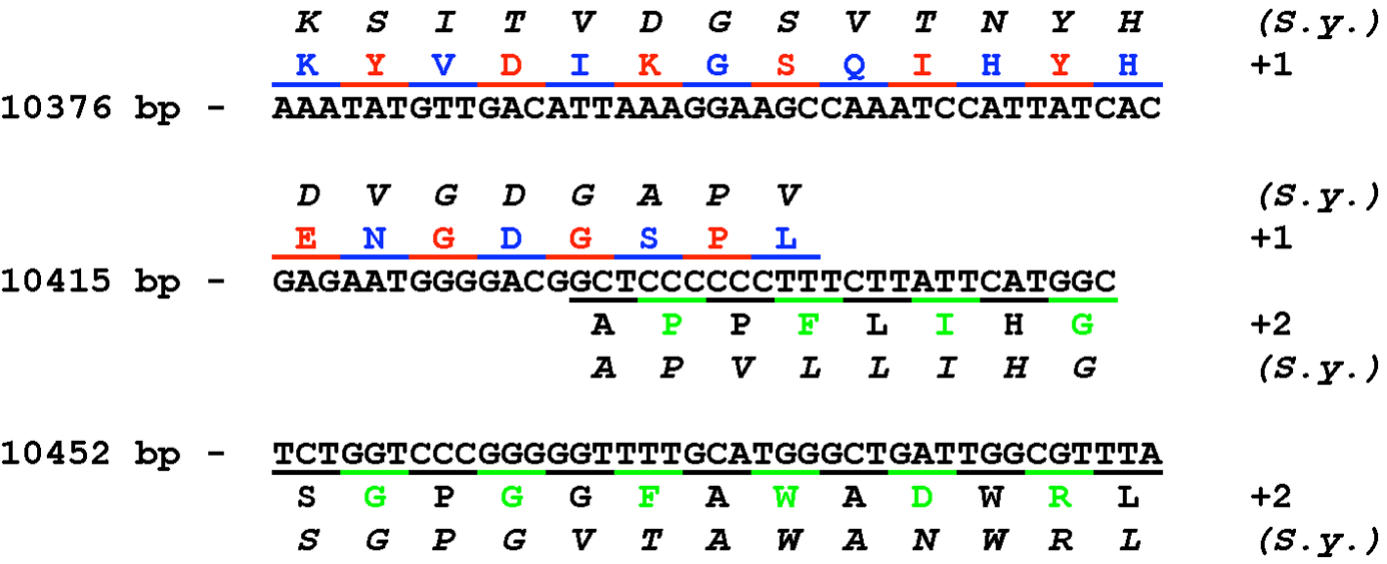
**Identification of a truncated HMSA-H downstream of *pheX***. A 115 bp stretch displays similarities in overlapping reading frames (+1 and +2) to HMSA-Hs as exemplified for MhpC from *Sphingobium yanoikuyae *(S.y.).

All enzymes for the complete mineralization of (alkyl-)phenols are encoded in the *phe*-operon with the exception of HMSA-DH and HMSA-H. It would be expected that *G. stearothermophilus *requires both since the organism is able to grow on phenol and methylphenols. As mentioned before, the compounds are metabolized via the hydrolytic route or the 4-oxalocrotonate route depending on the substitution. Interestingly, we identified a 115 bp stretch approximately 70 bp downstream of *pheX *that displays similarities to HMSA-Hs in two overlapping reading frames. This is illustrated in Figure 4 with a HMSA-H from *Sphingobium yanoikuyae *(GenBank ABM79791).

### ORFs in the vicinity of the *phe*-Operon

The DNA walk revealed four additional ORFs in the vicinity of the *phe*-gene cluster. No function in the phenol metabolism can be attributed to either of them. *Rep *encodes a plasmid replication initiator protein and *doc *encodes a death-on-curing protein. Both display the highest similarities to homologous from *Geobacillus kaustophilus *HTA426 plasmid pHTA426 (Doc: 92,9/94,3%; GenBank YP_147699; Rep: 57,7/68,8%; GenBank YP_145813). *OrfP *and *orfM *encode proteins of unknown functions. The latter is the only ORF located on the reverse strand.

### Transcriptional organization of the *phe*-genes

**Figure 5:**
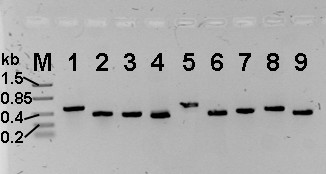
**Result of RT-PCR**. The intergenic spaces were amplified from a cDNA template to prove that the *phe*-genes including *fdx *are transcribed in one polycistronic mRNA. Lane 1, intergenic space *pheB/pheA1 *(including *pheH*)-expected size 512 bp; 2, *pheA1/pheA2*-365 bp; 3, *pheA2/fdx*-333 bp; 4, *fdx/pheR*-298 bp; 5, *pheR/pheD*-515 bp; 6, *pheD/pheF*-318 bp; 7, *pheF/pheE*-353 bp; 8, *pheE/pheC*-395 bp; 9, *pheC/pheX*-310 bp. The lane numbers also refer to the respective primer pairs listed in table 2 used for the amplification.

Bacterial genes belonging to a metabolic pathway are often grouped and transcribed in one polycistronic mRNA. This was examined for the *phe*-genes by reverse transcription-PCR (RT-PCR). The intergenic spaces of adjacent genes or in the case of *pheH *the intergenic space between *pheB *and *pheA1 *including *pheH *were amplified from a cDNA template. As shown in Figure 5, all *phe*-genes including *fdx *and the functionally divergent *pheX *are co-transcribed into a single 9.95 kb polycistronic mRNA. Therefore the *phe*-gene cluster can correctly be termed *phe*-operon. Moreover, the results indicate that phenol is metabolized via the *meta*-pathway since the cells were grown initially on minimal medium containing phenol as the sole carbon source prior to RNA extraction.

### Extrachromosomal localization

**Figure 6:**
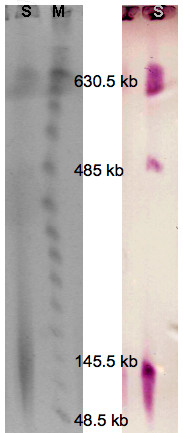
**PFGE (left) and Southern blot after hybridization with DIG-labeled probe (right)**. The megaplasmid pGGO1 was successfully separated from chromosomal DNA. Only weak bands representing the different plasmid conformations can be seen on the gel, which nevertheless generated strong signals on the blot. M, Lambda Ladder; S, sample.

It was suggested previously, that plasmid pGGO1 harbours the *meta*-pathway genes [10]. This was verified on a molecular level by pulsed field gel electrophoresis (PFGE) and Southern hybridization. The plasmid was separated from chromosomal DNA followed by hybridization with a digoxygenin (DIG)-labeled DNA-probe. Figure 6 shows the different conformations-open circular, supercoiled, linear-although the supercoiled form is rather weak. Nevertheless, all bands gave a clear hybridization signal. Size estimations based on these findings are vague but pGGO1 does not seem to exceed 150 kb.

## Discussion

Microorganisms can be found in almost any environmental niche due to their metabolic diversity. Often they are able to utilize toxic and xenobiotic compounds as energy sources. This can be exploited technically for bioremediation processes and has shown promising results in the past [17,18]. Under aerobic conditions specialized metabolic pathways usually break down these compounds enzymatically to intermediates of the Krebs cycle. *G. stearothemophilus *DSM6285 is able to grow on phenol and methyl-substituted phenols as a sole carbon source. Phenolic compounds are mineralized via the *ortho*- or the *meta*-pathway and can co-exist. This was also suggested for *G. stearothermophilus *[10]. The substrate specificity is not always distinct and can depend on the organism. However, methylphenols are degraded strictly via the *meta*-pathway [19] and a preference for phenol was also reported for certain pseudomonads [20].

We started a DNA walk from a previously identified 2.1 kb DNA fragment initially to isolate a C23O for biosensoric applications but continued to elucidate the organization of all *meta*-pathway genes. 15 ORFs were identified on a 20.2 kb fragment. The *phe*-operon represents the first complete *meta*-pathway-operon from thermophilic bacteria. It comprises eleven genes that are transcribed in one polycistronic mRNA. The arrangement differs from the organization of the *phe*-gene cluster from the closely related *G. thermoglucosidasius *[4]. All genes with the exception of *pheX *encode proteins associated with the phenol degradative *meta*-pathway. This includes catalytic enzymes as well as a regulatory protein and a ferredoxin for the reactivation of C23Os. Interestingly, neither a HMSA-DH nor a HMSA-H triggering the hydrolytic or the 4-oxalocrotonate branch of the *meta*-pathway seem to be present within the gene cluster or located in the near vicinity. Only a short fragment displaying similarities to HMSA-Hs in two overlapping reading frames was identified. This is surprising the more so as *G. stearothemophilus *is able to grow on phenol and 4-methylphenol that are converted via the 4-oxalocrotonate route as well as 2- and 3-methylphenol that are mineralized via the hydrolytic route [5]. Whether PheX represent a novel HMSA-H has to be examined in further experiments but seems unlikely from a current point of view. The amino acid sequence displays homologies to carboxylesterases and lipases, which, however, fall into the class of hydrolases, too. Moreover, lipases and HMSA-Hs share a consensus motif originally described for lipases that is also found within the PheX sequence [16]. Nevertheless, PheX displays no significant similarities to other HMSA-Hs including PheC from *G. thermoglucosidasius*. A direct role of esterases in the phenol metabolism is not known. No function could be attributed to OrfM and OrfP.

The *phe*-genes are organized in an operon as shown by RT-PCR. Grouping of associated genes facilitates the transcriptional regulation. Generally speaking, regulatory elements can be encoded within the operon under the control of the same promoter as the other structural genes or by one or more genes in the near vicinity. The transcriptional regulators of the *xyl*- and the *dmp*-genes from *P. putida *encoding the catalytic proteins of the *meta*-pathway are organized in a separate transcriptional unit [6,7]. Similar findings were reported for the *aph*-genes of *Comamonas testosteroni *[21]. In contrast, *pheR *is co-transcribed with the other *phe*-genes. Very little is published on the regulation of the degradation of phenolic compounds in gram-positive organisms. To our knowledge, this is the first time that a regulator of the phenol metabolism from a gram-positive bacterium was identified.

Using PFGE we were able to prove on a molecular level that the genes of the *meta*-pathway reside on megaplasmid pGGO1. The results confirm earlier findings based on phenotypic observations [10]. Specialized metabolic pathways are not necessarily a part of the basic bacterial metabolic repertoire but were rather acquired through horizontal gene transfer under selective pressure. Hence, a stable integration of the DNA into the genome is possible but often the genes are encoded on low-copy megaplasmids. Extrachromosomal elements as large as 1,683 kb have been reported [22]. Size estimations of pGGO1 based on the PFGE results were rather imprecise. Stable inheritance is often ensured by postsegregational killing systems also referred to as genetic addiction. Bacteriophage P1 is maintained as a stable low-copy plasmid in *E. coli *through a toxin-antitoxin system encoded by two genes termed *doc *(death on curing) and phd (prevent host death) [23]. More recently the *tasA*-*tasB *addiction system from plasmid pGI1 of *Bacillus thuringiensis *has been described [24]. The identification of *doc *suggests a similar mode of inheritance for pGGO1, although a gene encoding an antidot has not been isolated yet.

Sequencing of the whole plasmid would give a more detailed insight into the genetic organization of the *meta*-pathway in *G. stearothermophilus *and answer many open questions. The identification of the genes encoding a HMSA-H and HMSA-DH would be of particular interest. We will focus on a functional characterization in future work and elucidate the differences in the phenol metabolism between gram-negative organisms and thermophilic bacteria. The latter have received surprisingly low attention in comparison even though enzymes of thermophilic origin are prime candidates for new biotechnological applications [25].

## Conclusion

Despite the numerous publications concerning the degradation of aromatic compounds by gram-negative bacteria, very little is known about the genetic basis for the mineralization by gram-positive bacteria. In combination with the growing interest in enzymes of thermophilic origin this has prompted us to look more closely at the *meta*-pathway genes of *G. stearothermophilus*. The sequence of the operon and the deduced proteins show, that there seem to be no differences in the subsequent catalytic steps involved in the metabolization of phenol. Nevertheless, a detailed comparison of the enzymes with its mesophilic homologous will probably reveal structural aspects for the enhanced thermal stability. However, most intriguing is the identification of the first transcriptional regulator of *meta*-pathway genes in gram-positive bacteria. The moderate sequence homology on the one hand and since *pheR *is co-transcribed with the other *phe*-genes on the other hand suggest a variant regulatory mode which remains subject of further investigations.

## Methods

### Bacterial strains and growth conditions

*Geobacillus stearothermophilus *DSM6285 was obtained from the German Collection of Microorganisms and Cell Cultures (DSMZ). It was routinely grown at 55°C in minimal medium 458 recommended by the DSMZ containing 50 mM phenol as a sole carbon source. Phenol was replaced by other aromatic substances in the same molar concentration if applicable.

### Construction of libraries for PCR-based DNA walk

Total DNA from *G. stearothermophilus *was isolated as described elsewhere [26]. Four DNA libraries for the DNA walk were constructed according to the manufacturer's recommendations of the BD GenomeWalker Universal Kit (BD Biosciences Clontech) after digestion of the bacterial DNA with *Eco*RV, *Dra*I, *Pvu*II and *Ssp*I.

### Touchdown-PCR

PCR was performed with the Advantage Genomic Polymerase Mix (BD Biosciences Clontech) according to the manufacturer's instructions. Adaptor specific primers were included in the BD GenomeWalker Kit, gene-specific primers were obtained from Sigma-Genosys. The reaction protocol was optimized for the GeneAmp PCR System 9700 thermocycler (Applied Biosystems). The cycling conditions were as follows: 7 cycles at 94°C for 2 s, 70°C for 3 min; 32 cycles at 94°C for 2 s, 63°C for 3 min; final elongation at 67°C for 4 min. PCR products were analyzed on a 1% agarose gel and purified with the PCR Purification Kit (Jena Bioscience). Both strands were sequenced at least twice at MWG Biotech and analyzed with Vector NTI Advance 10 (Invitrogen).

### Pulsed field gel electrophoresis

PFGE was carried out with a LKB Pulsaphor apparatus with a hexagonal electrode array (LKB Bromma). Total DNA was prepared in agarose blocks according to a protocol for yeast, except that zymolase was substituted for lysozyme [27]. The sample plugs were loaded onto a 1%(w/v) agarose gel (Agarose MP, AppliChem), sealed with molten agarose and run in 0.5 × TBE buffer with 30 s pulses at 200 V for 22 h at 10°C. Lambda Ladder was purchased from Bio-Rad. The gel was stained with ethidium bromide (0.5 μg/mL) and visualized with a UV transilluminator.

### Labeling of DNA probes and Southern hybridization

DIG-labeled PCR fragments were used for Southern hybridization. The 904 bp *pheB *was amplified with GoTaq Flexi DNA Polymerase (Promega) under standard reaction conditions with primers *pheB_*1 (AAG GTT ACA TAT GGC TAT TAT GCG GAT CGG CAA GGC) and *pheB*_2 (AAT TCT CGA GTT ATG TCA GCG CCT TGA TGA ATG). DNA from PFGE gels was transferred to positively charged nylon membranes (porablot NY plus, Macherey-Nagel) by standard molecular biological methods as described elsewhere [28]. Labeling of the PCR product with DIG as well as hybridization of the probe to the blot and immunodetection was performed with the DIG High Prime DNA Labeling and Detection Starter Kit I (Roche) according to the manufacturer's recommendations.

### First strand cDNA-synthesis

Total RNA from *G. stearothermophilus *was isolated with the High Pure RNA Isolation Kit (Roche). To eliminate DNA contaminations one unit of DNAseI (Roche) was added per μg RNA and incubated for 15 min at 37°C followed by 15 min at 70°C. cDNA synthesis was performed with 1–3 μg RNA and 50 pmol random nonamer primer (NNN NNN NNN). The solution was incubated for 5 min at 70°C and cooled down on ice for primer annealing. 200 units MMLV reverse transciptase plus the provided reaction buffer (Promega) and 1 mM dNTPs were added to a total volume of 20 μL. Reverse transcriptase was omitted in the negative control. The sample was incubated for 30 min at 37°C followed by inactivation of the enzyme for 10 min at 70°C.

### Reverse transcription PCR

Multi-gene operons were analyzed by RT-PCR. Intergenic regions were amplified to verify, that the genes are transcribed in one polycistronic mRNA. cDNA and the cDNA negative control were used as templates. The latter ensured that no chromosomal DNA was carried over to the cDNA preparation. The PCR amplification was carried out with GoTaq Flexi DNA Polymerase (Promega) under standard reaction conditions. Oligonucleotides are listed in Table 2.

**Table 2:** Oligonucleotides for RT-PCR with the numbers indicating corresponding primer pairs

	Name	Sequence (5'-3')
1	*pheB*_down	TGA CGG AGT CAT TCA TCA AGG C
	*pheA1*_up	GCA AGA AGA GCA GCA GTG TCC AAC
2	*pheA1*_down	TTG CTA GCG GTG GCC GCA CAT CCT AAC
	*pheA2*_up	CGC ATT TGC AGT CAT GCC GTG TAT ATC C
3	*pheA2*_down	CAG GAA GAA GCC GAG CCA TTA ATC
	*Fdx*_up	CCT CGC CGC TAT CCA TTA CCT GCA C
4	*Fdx*_down	AGG AGG CGG ATG CGG AAT GTG CAA G
	*pheR*_up	AAG CAG CTG TCG GAA CCG TAA CC
5	*pheR*_down	CAA CCT TCC TAG CGT CAG TTA TC
	*pheD*_up	CCG CTT CTA TAA GGT GAT TAG C
6	*pheD*_down	GTG ATT CTT TCA GGC GCT CTT TCC G
	*pheF*_up	TTC CTG TGC TAA TGA CCG TAT ACC
7	*pheF*_down	ACG GAC TGA ACC AAT CAT GGA TGG A
	*pheE*_up	AGG AAC CGG CCA ATC CAT CTC CGT G
8	*pheE*_down	ATC GTA GCG CCA ATG CTT CCG GCA C
	*pheC*_up	CCT CTT GTA TCT TGT ATG CCT CTT CC
9	*pheC*_down	GTA TTC AAT CAC CCG GCC AAT TCT GTC G
	*pheX*_up	CCG CTT CTC CGC CAG AAG GAA GAT G

### Similarity search and sequence alignment

Similarity searches were performed with translated DNA sequences within the GenBank Peptide Sequence Database ("Blastx") using the BLAST algorithm [29]. Protein-sequence alignments are based on the BLOSUM62 substitution matrix [30] and the results are given in percent sequence identity and percent sequence similarity, respectively.

### HPLC analysis

HPLC analysis was performed on a Nucleosil 100-5 C18 column (CS Chromatographie Service) at 40°C using a Beckman Coulter System Gold equipped with a Triathlon autosampler and a Diode Array Detector Module 168. The compounds were separated with a 50/50 mixture of acetonitrile and acidified water (pH 3 with H_3_PO_4_) as the mobile phase at a flow rate of 0.5 mL/min.

## Abbreviations

4OD: 4-oxalocrotonate decarboxylase; 4OT: 4-oxalocrotonate tautomerase; AcDH: acetaldehyde dehydrogenase; C23O: catechol 2,3-dioxygenase; DIG: digoxygenin; HMA: 2-hydroxymuconic acid; HMSA: 2-hydroxymuconic acid-6-semialdehyde; HMSA-DH: HMSA dehydrogenase; HMSA-H: HMSA hydrolase; HOV: 4-hydroxy-2-oxovalerate; HOVA: HOV aldolase; OC: 4-oxalocrotonate; OE: 2-oxopent-4-dienoate; OEH: OE hydratase; ORF: open reading frame; PH: phenol hydroxylase; RT-PCR: reverse transcription-PCR

## Authors' contributions

MR and WH conceived the study, participated in its design and revised the manuscript critically. UKJ initiated the PCR-based DNA walk and constructed the libraries. MZ participated in the PFGE in general, the sample preparation in particular and edited the manuscript. BO carried out the DNA walk in the latter stages, performed the PFGE and Southern hybridization, the RT-PCR, the *in silico *work and wrote the manuscript. All authors read and approved the final manuscript.
